# Investigation of Tin Oxide Atomic Layer Deposition on the Performance of Evaporated Perovskite Solar Cells

**DOI:** 10.1002/smll.75573

**Published:** 2026-09-07

**Authors:** Bhavya Rakheja, Adam Hultqvist, Maart Van Druenen, Pontus Törnblom, Fredrik Larsson, Jörgen Olsson, Carl Hägglund, Evelyn Johannesson, Laurent Duda, Håkan Rensmo, Erik M. J. Johansson, Erik Wallin, Tobias Törndahl

**Affiliations:** ^1^ Department of Materials Science and Engineering Solar Cell Technology Uppsala University Uppsala Sweden; ^2^ First Solar European Technology Center Uppsala Sweden; ^3^ Department of Physics and Astronomy X‐ray Photon Science Uppsala University Uppsala Sweden; ^4^ Department of Chemistry – Ångström Laboratory Physical Chemistry Uppsala University Uppsala Sweden

**Keywords:** atomic layer deposition, perovskite solar cells, tin oxide, transport layers

## Abstract

Although tin oxide (SnO_x_) layers grown using atomic layer deposition (ALD) are routinely employed in p‐i‐n perovskite solar cells (PSCs), the correlation between ALD process parameters, SnO_x_ film properties, and PSC performance remains insufficiently investigated. This work examines the role of co‐reactants, H_2_O and H_2_O_2_, and their doses in influencing SnO_x_ material properties and the performance of evaporated p‐i‐n PSCs. H_2_O_2_‐SnO_x_ exhibits higher film density and higher electron affinity compared to H_2_O‐SnO_x_; however, PSCs incorporating H_2_O‐SnO_x_ demonstrate superior performance. The performance of PSCs containing H_2_O_2_‐SnO_x_ is limited by an undesired chemical interaction between H_2_O_2_ and the underlying structure, which can be mitigated by employing a thin layer of H_2_O‐SnO_x_ prior to the deposition of H_2_O_2_‐SnO_x_ on top of the perovskite/C_60_ structure. Furthermore, the H_2_O dose used during the ALD of SnO_x_ substantially affects PSC performance. High H_2_O dose‐SnO_x_ yields the highest power conversion efficiency (19%), whereas low H_2_O dose‐SnO_x_ results in the lowest efficiency (11.3%). Low H_2_O dose‐SnO_x_ exhibits low film density and excess ligand impurities, which can act as charge traps and introduce transport barrier when incorporated into PSCs. Therefore, high H_2_O doses are indispensable during ALD of SnO_x_ to achieve efficient PSC performance.

## Graphical abstract

Impact of co‐reactants and their doses utilized during the atomic layer deposition of SnO_x_ on perovskite/C_60_ structures on the performance of p‐i‐n perovskite solar cells.

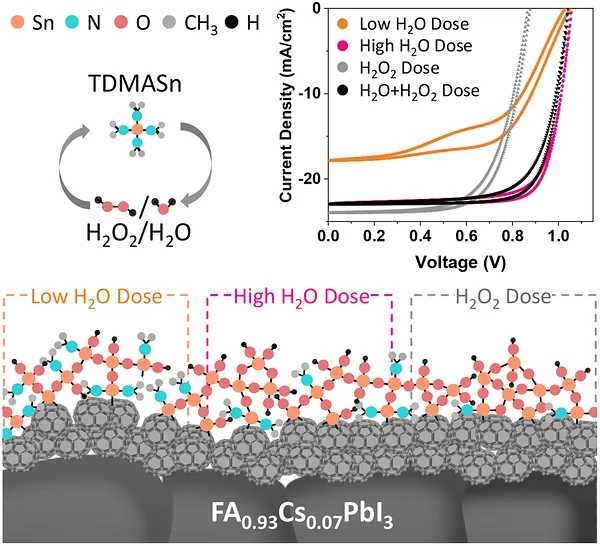

## 1 Introduction

Metal halide perovskite (MHP) technology has witnessed swift progress during the last decade in the field of photovoltaics. Their use has revolutionized the field by achieving power conversion efficiencies (PCEs) comparable to the well‐established silicon solar cell technology [1]. The phenomenal success witnessed by MHPs is a result of their outstanding optoelectronic properties such as high absorption coefficient [2], long charge carrier diffusion length [3], and high charge carrier mobilities [4]. The combination of these properties makes MHPs one of the best candidates for the absorber layer in solar cell structures. Besides, perovskite solar cells (PSCs) in conjunction with silicon solar cells in a tandem structure have demonstrated efficiencies surpassing their standalone parts [5, 6, 7]. However, PSCs still underperform in terms of long‐term stability compared to the commercialized technologies. Further, the top PCE values are limited to laboratory‐scale solution‐processed PSCs, and decrease significantly when scaled‐up to large‐areas [8]. Therefore, research on PSCs fabricated using industry‐compatible deposition techniques, such as evaporation, is indispensable for the commercialization of PSCs.

Besides the MHP absorber layer, PSCs are also comprised of other thin‐film layers that play a critical role in defining the solar cell performance. In particular, charge transport layers (CTLs), that facilitate the transport of generated charge carriers, need to provide charge selectivity, have a good energetic alignment with the MHP layer as well as form good interfaces containing minimal defects with the adjacent layers. When it comes to the electron transport layer (ETL), tin oxide (SnO_x_) is one of the most suitable candidates owing to its high optical transparency, low temperature processability, and favorable conduction band alignment with the MHPs [9]. Currently, all top efficiency p‐i‐n PSCs contain a bilayer of fullerene (C_60_) or Phenyl‐C61‐butyric acid methyl ester (PCBM) and SnO_x_ grown using atomic layer deposition (ALD) [7, 10, 11]. Here, C_60_ or PCBM efficiently extracts the generated electrons and SnO_x_ protects the underlying structure from the subsequent sputtering step of transparent‐conductive oxide (TCO). Moreover, SnO_x_ acts as a permeation barrier against the exchange of species to and from the MHP layer, thus improving its long‐term stability [12, 13].

Owing to the tunable nature of ALD, both material and electronic properties can be tailored by modifying the deposition parameters. The properties of SnO_x_ grown using ALD are known to be dependent on the deposition temperature [14] and the choice of co‐reactant [15, 16]. Previously, the effect of SnO_x_ layers grown using different co‐reactants, including H_2_O_2_, ozone, oxygen plasma, has been studied on the performance of n‐i‐p PSC structures, where perovskite is deposited on top of the SnO_x_ layer [15, 16, 17]. The employment of a H_2_O_2_ co‐reactant instead of H_2_O during the ALD of SnO_x_ has been demonstrated to result in films with fewer oxygen‐vacancies [16]. As a consequence, the utilization of the H_2_O_2_ co‐reactant was reported to be beneficial in improving the electron extraction properties of the SnO_x_ layer and, in enhancing the PSC performance. Further, Hu et al. also compared the effect of different co‐reactants, including ozone, oxygen plasma and H_2_O, utilized during the ALD of SnO_x_ on the performance of n‐i‐p PSCs. Here, the choice of co‐reactant during the ALD process of SnO_x_ was reported to be crucial in determining the interfacial dipole between SnO_x_ and the adjacent perovskite layer [15].

So far, the investigation of co‐reactant choice during the ALD of SnO_x_ has mainly been limited to n‐i‐p PSCs. Even though SnO_x_ grown using ALD is widely employed in p‐i‐n PSCs, a clear understanding of the influence of deposition parameters on the material properties, and their correlation with the solar cell performance, is lacking. From the perspective of a solar cell, it is of utmost importance that SnO_x_ has a favorable conduction band alignment with the adjacent C_60_ layer in p‐i‐n structures. Besides, minimizing bulk and interface defects is pivotal in mitigating power losses in PSCs. Therefore, this study establishes a detailed understanding of the importance of choosing ALD process parameters toward achieving optimal solar cell performance. Considering the relevance of industry‐compatible deposition methods for the commercialization of PSCs, we have specifically studied the SnO_x_ ALD process for its employment in PSCs comprising evaporated perovskite absorbers.

In this work, the role of co‐reactants is investigated in‐depth for the ALD process of SnO_x_. Here, tetrakis(dimethylamino)tin(IV) (TDMASn) is utilized as the metal precursor and the effect of co‐reactants, i.e., water (H_2_O) and hydrogen peroxide (H_2_O_2_), is studied. First, the focus is placed on how H_2_O co‐reactant dose and deposition temperature influence the properties of SnO_x_. Then the question of how relationships and properties change when H_2_O_2_ rather than H_2_O co‐reactant is used for film growth, is addressed. Finally, SnO_x_ films prepared at a deposition temperature of 100°C using H_2_O and H_2_O_2_ as co‐reactants are incorporated into evaporated (Cs, FA)PbI_3_ and (Cs, FA)Pb(I,Br)_3_ PSC structures to characterize their impact on the solar cell performance.

## 2 Results

### 2.1 Physical Properties and Growth Behavior

Figure 1a presents Sauerbrey mass change per cycle values obtained using quartz crystal microbalance (QCM) as a function of H_2_O dose at a deposition temperature of 100°C. The obtained QCM curves and mass change values are additionally provided in Figure S1 and Table S1, respectively. Here, low, medium and high doses were measured to correspond to an evaporation of 0.006 mg/cycle, 0.061 mg/cycle and 0.258 mg/cycle of H_2_O, respectively. The growth per cycle (GPC) increases from 74 ng/cm^2^ for the low H_2_O dose‐SnO_x_ to 83 ng/cm^2^ for the high H_2_O dose‐SnO_x_. Figure 1b shows the density and thickness values obtained using x‐ray reflectivity (XRR) for SnO_x_ films grown using different H_2_O doses on quartz substrates. Measured and simulated XRR curves are presented in Figure S2, and the fitted density and thickness values are given in Table S1. The density value increases from 4.5 g/cm^3^ to 5.0 g/cm^3^ between SnO_x_ films prepared using low and high doses of H_2_O. Furthermore, the film thickness increases from 113 nm for the low H_2_O dose‐SnO_x_ film to 129 nm for the high H_2_O dose‐SnO_x_ film for a total 1000 ALD cycles. Moreover, x‐ray fluorescence (XRF) measurements conducted on SnO_x_ films demonstrate an increase in Sn Kα counts as a function of H_2_O dose (Figure S3). Thus, the combination of QCM, XRR and XRF results confirms an increase in both film density and GPC value as a function of increasing H_2_O dose.

**FIGURE 1:**
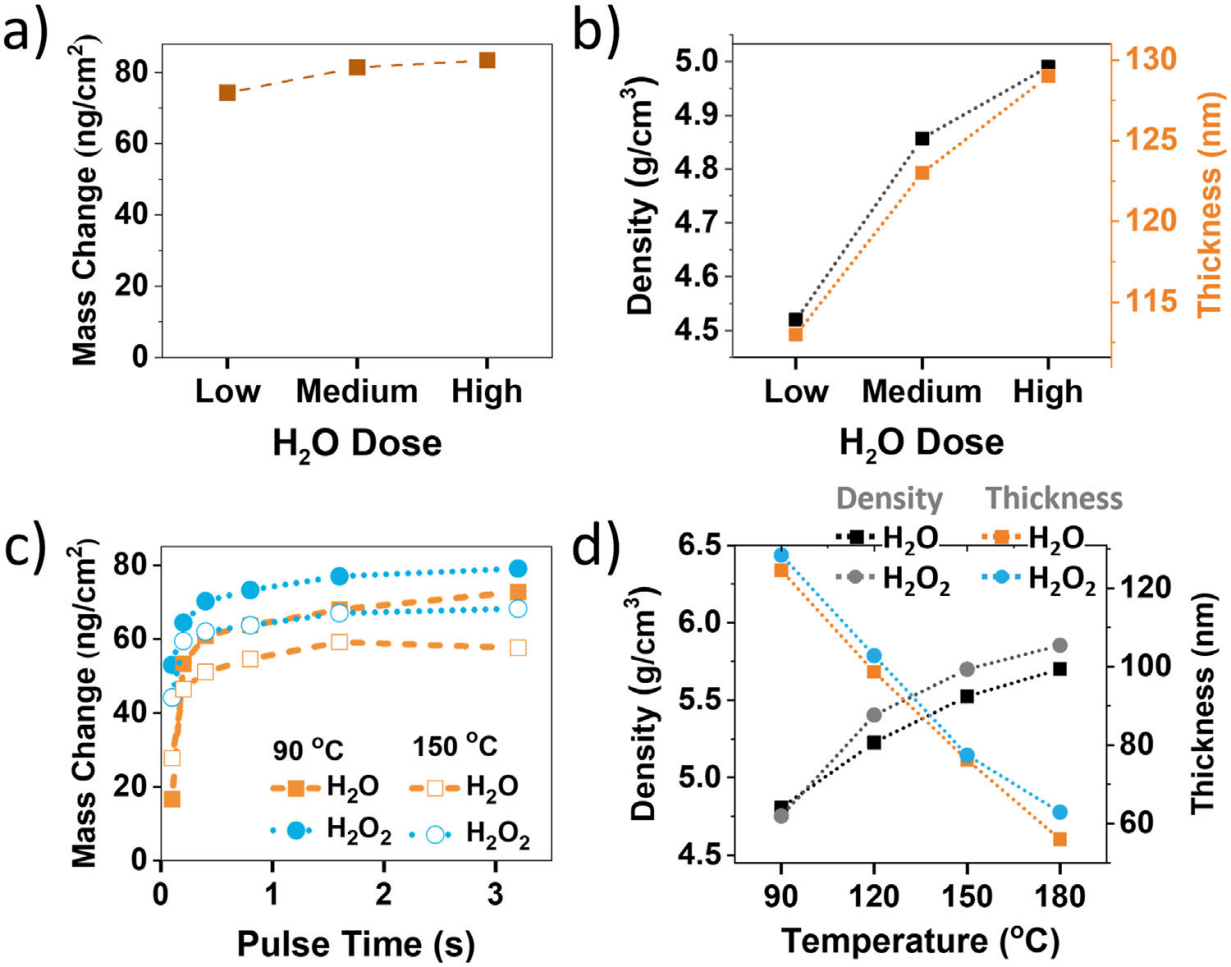
(a) QCM mass change per cycle and, (b) density and thickness values obtained using XRR for 1000 ALD cycles of SnO_x_ on quartz substrates grown using different H_2_O doses at 100°C. Comparison between H_2_O and H_2_O_2_ co‐reactants for SnO_x_ process at different deposition temperatures using (c) QCM mass change per cycle and, (d) density and thickness values obtained using XRR for 1000 ALD cycles of SnO_x_ on quartz substrates.

Figure 1c shows the saturation curves for SnO_x_ process as a function of co‐reactant, H_2_O and H_2_O_2_, pulse lengths at deposition temperatures of 90°C and 150°C, obtained using medium doses of each. The corresponding saturation curves for TDMASn pulse length and N_2_ purge lengths can be found in Figure S4. The GPC value is found to be higher at 90°C in comparison to the value at 150°C, irrespective of the co‐reactant choice. However, the corresponding GPC value is higher in the case of H_2_O_2_‐SnO_x_ for the same pulse length at both temperatures. Figure 1d presents the density and thickness values of SnO_x_ films grown using H_2_O and H_2_O_2_ as co‐reactants at deposition temperatures of 90°C, 120°C, 150°C, and 180°C. Measured and simulated XRR curves can be found in Figure S5, with the fitted density and thickness values listed in Table S2. All the SnO_x_ films are found amorphous in nature without the presence of any reflections in their respective x‐ray diffraction (XRD) pattern (Figure S5). The density of the films increases with an increase in the deposition temperature, while the thickness decreases, regardless of the utilized co‐reactant during the SnO_x_ process. H_2_O_2_‐SnO_x_ films have higher density values compared to H_2_O‐SnO_x_ films at all deposition temperatures except for 90°C, where the values are comparable. Simultaneously, films grown using the H_2_O_2_ co‐reactant also result in higher values of thickness across all deposition temperatures. In parallel, spectroscopic ellipsometry (SE) and transmittance measurements were conducted on all SnO_x_ samples to analyze their optical properties, especially the bandgap values. The details concerning SE and bandgap calculations are provided in the supplementary information. The transmittance of SnO_x_ films prepared using both H_2_O and H_2_O_2_ co‐reactants is found to decrease as a function of the deposition temperature. Overall, the bandgap value decreases from 3.27 to 2.63 eV for H_2_O‐SnO_x_ films and from 3.21 to 2.59 eV for H_2_O_2_‐SnO_x_ films with an increase in the deposition temperature from 90°C to 150 °C (Figure S6). Whereas further increase in the deposition temperature to 180°C, results in a small increase in the bandgap value to 2.76 eV for H_2_O‐SnO_x_ and 2.64 eV for H_2_O_2_‐SnO_x_ films. Overall, the choice of co‐reactant and co‐reactant dose display only a minor effect on the bandgap values (refer to Table S3 and Table S4).

### 2.2 Chemical Properties

The chemical composition and stoichiometry of the SnO_x_ films grown using different doses of H_2_O at 100°C were studied using x‐ray photoelectron spectroscopy (XPS) and Resonant inelastic x‐ray scattering (RIXS). XPS spectra of O 1s, Sn 3d_5/2_, N 1s and C 1s core‐levels and valence band (VB) are shown in Figure 2a–e, and the RIXS maps of N K‐edge are presented in Figure 2f. All of the XPS spectra are normalized with respect to the integrated area of Sn 3d_5/2_ core level and the original spectra are presented in Figure S7. The O 1s spectra, shown in Figure 2a, comprise two peaks at binding energy values of ∼531.15 eV and ∼532.55 eV for the medium and high H_2_O dose‐SnO_x_, while these peaks are shifted by 0.11 eV toward higher binding energy values for the low H_2_O dose‐SnO_x_ sample. The peak at the lower binding energy (∼531.2 eV) is associated with the presence of Sn‐O bonds in the SnO_x_ film, whereas the peak at higher binding energy (∼532.6 eV) is often attributed to a combination of Sn‐OH and a minor contribution from carboxyl species [18, 19, 20, 21]. The presence of carboxyl species can also be seen in the corresponding C 1s core level spectra at ~289.6 eV, shown in Figure 2d. The contribution from the carboxyl component could be a post deposition effect or originate due to a partial oxidation of Sn‐precursor products [19]. The carboxyl component present in the C 1s spectra does not vary across samples grown using different doses of H_2_O. Thus, the variations observed in the integrated area of O 1s spectra, as a function of H_2_O dose, are linked to the changes in the concentration of Sn‐OH and/or Sn‐O bonds. The ratio [O_Sn‐OH_]/[O_Sn‐O_] (corresponding to Sn‐OH/Sn‐O bonds) decreases from 0.43 for low H_2_O dose‐SnO_x_ to 0.36‐0.37 for medium and high H_2_O dose‐SnO_x_. Note that the brief air exposure prior to the acquisition of XPS data could minorly influence the absolute value of this ratio; however, the trends observed across samples in the ratio would still apply. Sn 3d_5/2_ core‐level (Figure 2b) demonstrates a single peak related to Sn^4+^ in SnO_x_, centered at the binding energy value of ~487.4 eV for medium and high H_2_O dose‐SnO_x_ but shifted about 0.07 eV for the low H_2_O dose‐SnO_x_ sample. The intensity of the Sn 3d_5/2_ peak measured on SnO_x_ films increases 21% with an increase in H_2_O dose from low to high (shown in Figure S7). As is also corroborated by XRF measurements, it is therefore concluded that high H_2_O dose‐SnO_x_ comprises the highest concentration of Sn among the three samples. Furthermore, the VB spectra (Figure 2e) demonstrate a small shift of 0.17 eV in valence band maximum (VBM) position with an increase in H_2_O dose. The VBM shifts from 3.89 eV for low H_2_O dose‐SnO_x_ to 3.72 eV for high H_2_O‐SnO_x_. The 0.17 eV shift is comparable to the shifts observed in the O 1s and Sn 3d core‐level spectra, and hence consistent with a rigid band shift.

**FIGURE 2:**
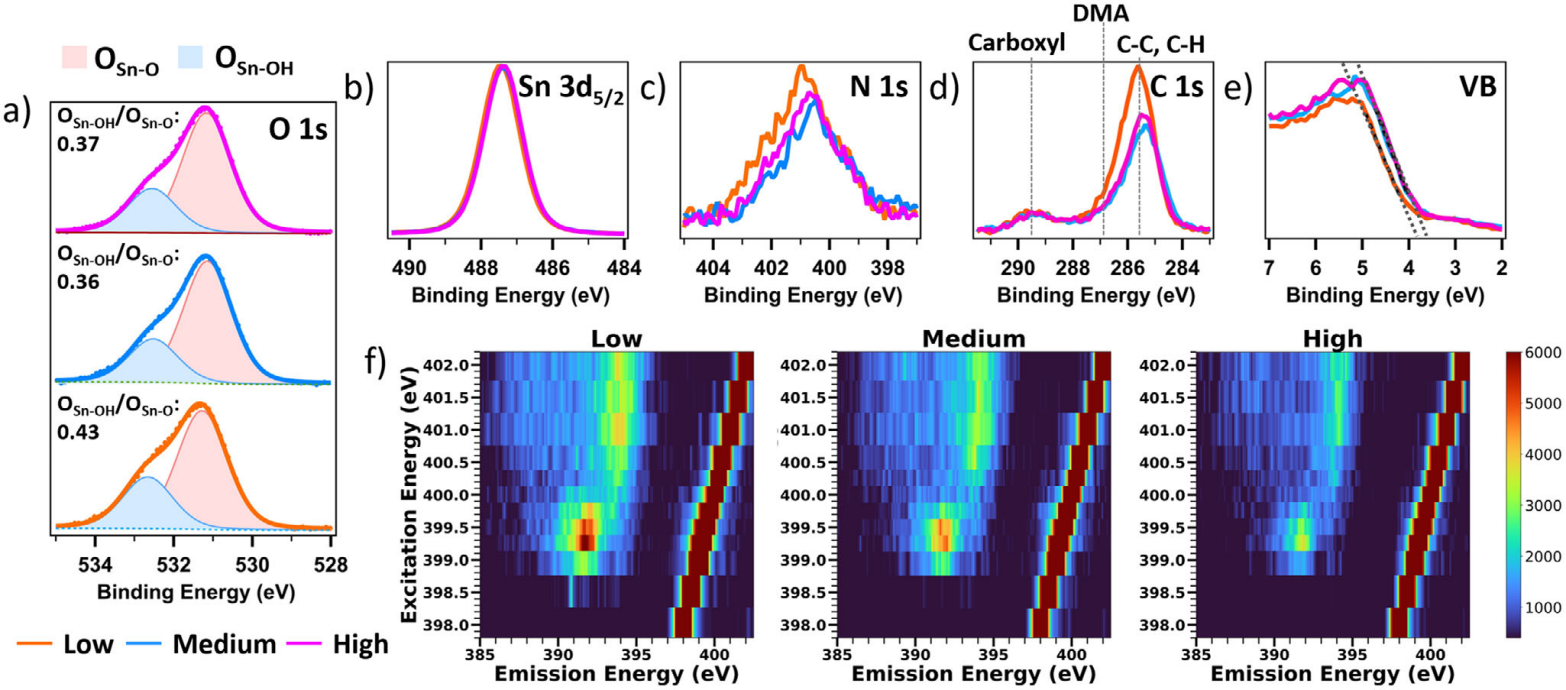
XPS spectra of (a) O 1s, (b) Sn 3d_5/2_, (c) N 1s and (d) C 1s core‐levels and (e) valence band (VB) obtained using an Al Kα x‐ray source and (f) RIXS maps of N K‐edge of SnO_x_ films grown using different doses of H_2_O at 100°C.

Finally, N 1s and C 1s core‐level spectra shown in Figure 2c,d provide insights on the amount of residual dimethylamine (DMA) ligand in the SnO_x_ films. All samples exhibit residual DMA ligand impurities, regardless of the utilized H_2_O dose and the results indicate that the nitrogen content in the low H_2_O dose‐SnO_x_ is slightly higher. However, limited signal‐to‐noise ratio of these XPS core‐levels require complementary measurements to better assess the amount of DMA impurities within different films. To complement the XPS results, RIXS measurements were performed in parallel to probe the entire bulk of SnO_x_ and reduce the effects of surface contamination by air exposure. Figure 2f displays RIXS N K‐edge maps for SnO_x_ films, selected N K‐edge RIXS data is provided in Figure S8. In this soft x‐ray regime, the RIXS signal is bulk‐sensitive and reflects the entire film thickness (100‐130 nm). The RIXS N K‐edge features originate from the residual DMA ligands in the film. Although the spectra are noisy due to a low nitrogen concentration, a clear trend depending on the H_2_O dose can be observed. The nitrogen signal and, thus, the corresponding DMA content decreases with an increase in H_2_O dose. Moreover, the similarity in the line shapes across the three samples suggests a common nitrogen species, most likely DMA. We also note that the O K‐edge RIXS (Figure S9) did not display any major variations among the samples prepared with low, medium and high H_2_O doses, indicating that the nitrogen concentration does not significantly perturb the oxygen electronic structure of SnO_x_. Overall, XPS and RIXS analysis provide direct evidence that high H_2_O dose‐SnO_x_ comprises a lower impurity content and a higher concentration of Sn within a given sample volume compared to low and medium H_2_O dose‐SnO_x_.

The O 1s and Sn 3d_5/2_ spectra recorded on SnO_x_ films prepared using H_2_O and H_2_O_2_ at deposition temperatures of 90°C, 120°C, 150°C and 180°C are presented in Figure 3. As discussed above, O 1s spectra consist of two components, representing Sn‐O bonds at the lower binding energy value (O_Sn‐O_) and Sn‐OH at the higher binding value (O_Sn‐OH_). The two contributions are indicated in the O 1s spectra shown in Figure 3a, with further details provided in Figure S10. For both co‐reactants, the higher binding energy component (O_Sn‐OH_) in the O 1s spectra reduces with an increase in the deposition temperature. Furthermore, the Sn 3d_5/2_ core‐level spectra (Figure 3b) demonstrate the emergence of an additional component at an energy separation of 0.6 eV with respect to the main Sn^4+^ peak as the deposition temperature is increased. This component can be attributed to Sn^2+^ as per the previous reports [22, 23]. The Sn 3d_5/2_ and O 1s core‐levels shift toward lower binding energy values upon increasing the deposition temperature, while the energy difference between the two core‐levels stays the same (43.8 eV). These observations imply a shift in the position of Fermi level toward the VBM.

**FIGURE 3:**
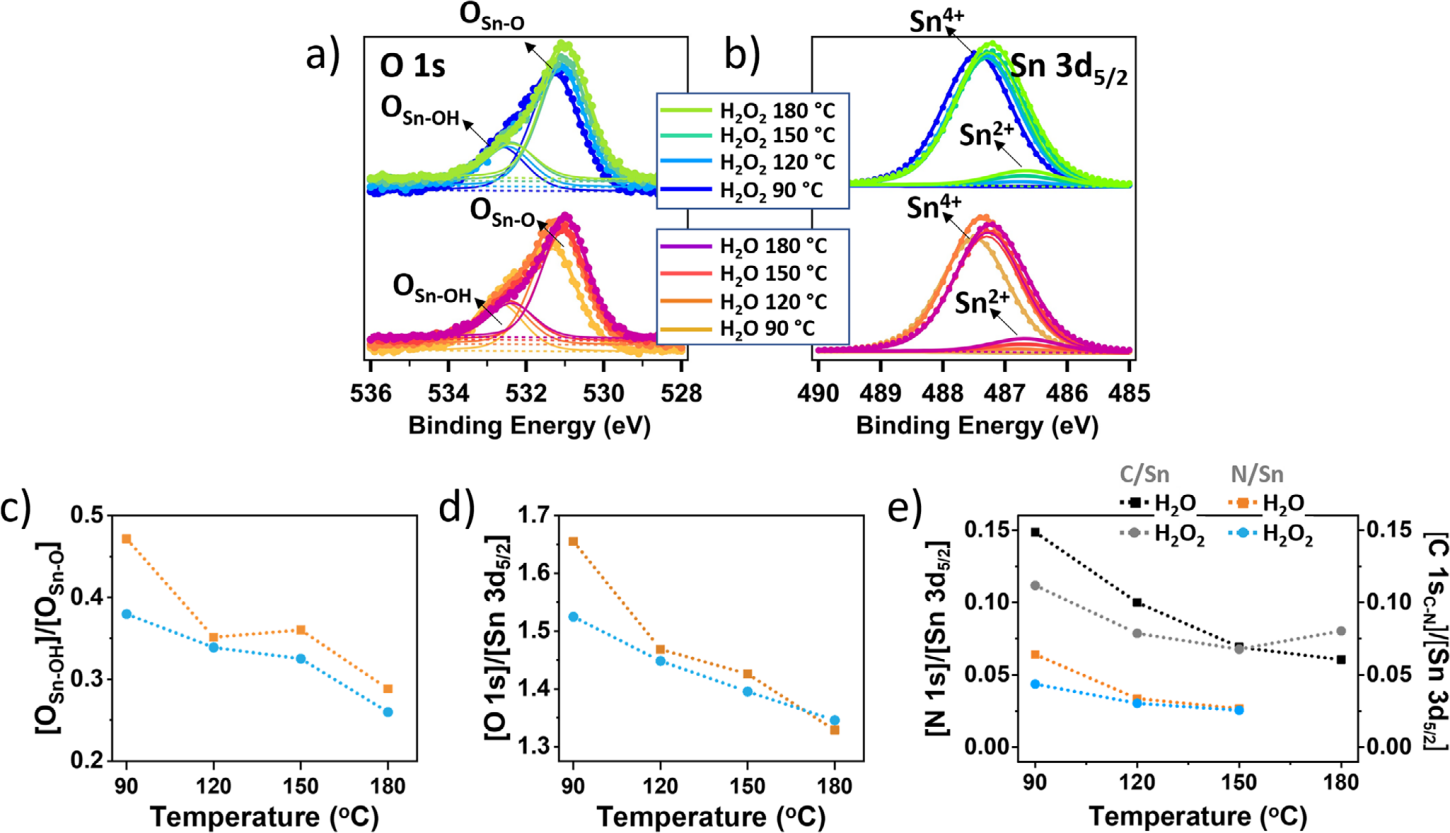
XPS spectra of (a) O 1s and (b) Sn 3d_5/2_ core‐levels acquired using an Al Kα x‐ray source, together with the corresponding concentration ratios of (c) O 1s components, (d) [O 1s]/[Sn 3d_5/2_], (e) [N 1s]/[Sn 3d_5/2_] and [C 1s (C‐N)]/[Sn 3d_5/2_] for SnO_x_ films grown using H_2_O and H_2_O_2_ co‐reactants at different deposition temperatures.

The ratio between the integrated areas of components, O_Sn‐OH_ and O_Sn‐O_, is presented in Figure 3c. Here, a gradual reduction in the Sn‐OH (O_Sn‐OH_) component as a function of deposition temperature can be observed. Interestingly, SnO_x_ films prepared using H_2_O_2_ exhibit a lower percentage of Sn‐OH bonds compared to the films grown using H_2_O at all deposition temperatures. Moreover, the overall [O]/[Sn] ratio, calculated using the total integrated areas of O 1s and Sn 3d_5/2_ core‐levels, also decreases with an increase in the deposition temperature (Figure 3d), which is in line with former studies [24]. The difference in the stoichiometry between the films prepared using H_2_O and H_2_O_2_ is more prominent at lower deposition temperatures, while the values become similar at higher deposition temperatures. Figure 3e presents the [C]/[Sn] and [N]/[Sn] elemental concentration ratios, calculated from the C‐N component of the C 1s and N 1s spectra shown in Figure S10. These ratios indicate that the concentration of residual DMA impurities decreases with increasing deposition temperature, in good agreement with a former study [25]. Furthermore, particularly at low deposition temperatures, the [C]/[Sn] and [N]/[Sn] ratios are lower for SnO_x_ films grown using H_2_O_2_ than for those grown using H_2_O. The observation suggests a more efficient ligand removal during SnO_x_ growth when H_2_O_2_ is employed as the co‐reactant.

### 2.3 Electrical Characterization

SnO_x_ films were integrated into metal‐oxide‐semiconductor (MOS) structures comprising aluminum as the metal electrode, and n‐doped silicon as the semiconductor to evaluate its electronic properties. *J–V* characteristics of MOS structures containing SnO_x_ grown using low H_2_O dose, high H_2_O dose and high H_2_O_2_ dose at a deposition temperature of 100°C are presented in Figure 4a. Application of a positive gate bias creates an accumulation region on the silicon side close to the SnO_x_ layer. In this case, the voltage drop mainly occurs across the SnO_x_ layer and the electric field (*E*) across the SnO_x_ can be easily determined if the thickness of the SnO_x_ layer is known. The current density (*J*) across the SnO_x_ layer can be described using Equation 1, as follows [26]:

(1) $$J=A^{*}\;T^{2}exp\lfloor \frac{-q}{kT}\left(\varphi_{B}-\sqrt{\frac{qE}{4\pi \varepsilon_{r}\varepsilon_{0}}}\right)\rfloor ,\;\;A^{*}=\frac{4\pi k^{2}m^{*}}{h^{3}}\;$$

**FIGURE 4:**
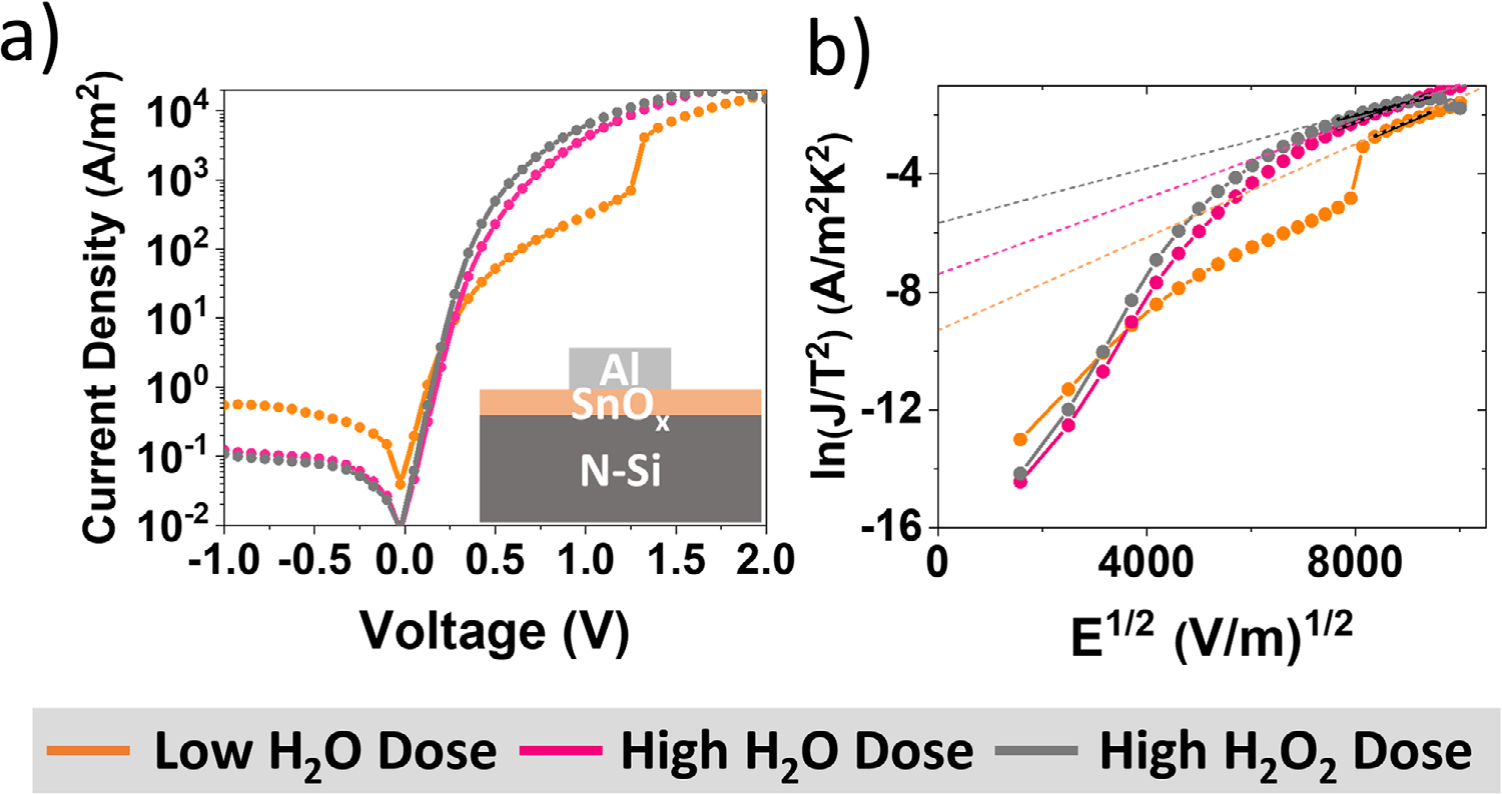
(a) Current‐voltage and (b) Schottky emission characteristics of MOS devices containing SnO_x_ prepared using low H_2_O dose, high H_2_O dose and high H_2_O_2_ dose at 100°C.

Here, *T* is the temperature in Kelvin, *q* is the elementary charge, φ_*B*_ is the barrier height, *k* is the Boltzmann constant, ε_*r*_ is the dielectric constant of the SnO_x_ layer and ε_0_ is the permittivity of free space. A*is the Richardson constant, which depends on the effective mass (m*) of charge carriers in the material. The y‐axis intercept (*b*) of the plot between *ln(J/T^2^)* and *(E)^1/2^* is linked to the barrier height between the conduction band of SnO_x_ and the Fermi level of silicon by Equation S1. Thus, the electron affinity of SnO_x_ can be estimated if the work‐function of the utilized silicon is known. Figure 4b presents the plot between *ln(J/T^2^)* and *(E)^1/2^* constructed for MOS structures containing different SnO_x_ films to find their respective electron affinity values. The obtained electron affinity values for low H_2_O dose‐SnO_x_, high H_2_O dose‐SnO_x_, and high H_2_O_2_ dose‐SnO_x_ are 3.70, 3.80 and 3.86 eV, respectively. Thereby, the utilized co‐reactant and its respective dose influence the electron affinity of SnO_x_. Furthermore, *J–V* characteristics of MOS structure containing low H_2_O‐dose SnO_x_ demonstrate the lowest value of current density among the three structures, thus indicating that low H_2_O dose‐SnO_x_ contributes a higher value of series resistance compared to high H_2_O dose‐SnO_x_, and high H_2_O_2_ dose‐SnO_x_.

In order to access the nature and concentration of the defect states, *C–V* measurements were also conducted on MOS structures (shown in Figure S11). It was only possible to obtain MOS‐like *C–V* characteristics for low H_2_O dose‐SnO_x_, likely linked to its higher barrier height and resistivity compared to high H_2_O‐dose‐SnO_x_, and high H_2_O_2_ dose‐SnO_x_. The existence of a hysteresis in the *C–V* characteristics of low H_2_O dose‐SnO_x_ layer indicates the presence of trapped oxide charges. The concentration of these trapped oxide charges was found to be approximately 1 × 10^11^ cm^−2^. The details concerning the calculations of trapped oxide charges can be found in the Supplementary Information. The *C–V* characteristics of high H_2_O dose‐SnO_x_ and high H_2_O_2_ dose‐SnO_x_ films are free from any hysteresis, indicating the absence of trapped charges.

### 2.4 Solar‐Cell Analysis

ITO/PTAA/perovskite/C_60_/SnO_x_/indium‐zinc‐oxide/Ag structures were fabricated to study the influence of SnO_x_ on the PSC performance. Here, perovskites were deposited using evaporation and the effect of SnO_x_ was studied on PSCs containing (Cs, FA)Pb(I,Br)_3_ and (Cs, FA)PbI_3_ perovskite absorbers. The details concerning perovskite deposition and solar cell fabrication are provided in the supplementary information. Even though SnO_x_ films prepared at high deposition temperatures comprise less defects and higher density values, they cannot be employed in p‐i‐n PSCs as MHPs are extremely temperature‐sensitive. Besides, the reduced transmittance of SnO_x_ at higher deposition temperatures is unsuitable for its employment in semi‐transparent PSCs. Therefore, a deposition temperature of 100°C was chosen for this part of the study.

Initially, the effect of the number of ALD cycles of SnO_x_ was evaluated on (Cs, FA)Pb(I,Br)_3_ PSCs, as shown in Figure S12. PSCs containing 80 cycles of SnO_x_ demonstrate a large variation in PCE values, with the best‐performing PSC having a PCE value of 14%. Here, the PSC performance is majorly limited by a low value of shunt resistance, suggesting that 80 cycles of SnO_x_ results in films that are too thin to protect the underlying perovskite/C_60_ structure from the subsequent sputtering process. In contrast, PSCs containing 130 and 180 cycles of SnO_x_ exhibit a narrower spread in PCE values, where the best‐performing PSCs of each kind display PCE values of 15.6% and 15.9%, respectively. PSCs comprising 230 cycles of SnO_x_ display a comparable PCE value of 15.5% for the best‐performing cell. However, the observed spread among the PCE values and the difference between the reverse and forward scan PCE values are large. These performance losses potentially originate due to an increase in the value of series resistance, as it increases with an increase in the thickness (number of ALD cycles). Based on these observations, 180 cycles were deemed close to optimum for the incorporation of SnO_x_ layer in PSCs.

Figure 5a,b compare the effect of H_2_O dose utilized during the ALD of SnO_x_ on the current‐voltage (*J–V*) characteristics, and PCE values of (Cs, FA)PbI_3_ PSCs, respectively. Here, box plots are constructed using the PCE values acquired on 16 cells of each variation and *J–V* characteristics are presented for the best‐performing cell of each. The PCE values of the best performing PSCs containing low H_2_O dose‐SnO_x_, medium H_2_O dose‐SnO_x_, and high H_2_O dose‐SnO_x_ are 11.3%, 18.7% and 19.0%, respectively. Thus, it is concluded that the PCE value of (Cs, FA)PbI_3_ PSCs improves with an increase in the utilized H_2_O dose during the SnO_x_ ALD. PSCs containing low H_2_O dose‐SnO_x_ demonstrates an s‐shaped *J–V* characteristics. Such a *J–V* shape typically represents the existence of a barrier in the transport of generated charge carriers between the two layers. Besides, PSCs containing low H_2_O dose‐SnO_x_ results in lower values of the short‐circuit current (*J_SC_*) and fill factor (*FF*) compared to PSCs containing medium or high H_2_O dose‐SnO_x_, as shown in Figure S13. On the contrary, the open‐circuit voltage (*V_OC_*) increases only minorly with an increase in the H_2_O dose utilized during the SnO_x_ process. The EQE curves shown in Figure 5c for PSC employing low, medium, and high H_2_O dose‐SnO_x_ demonstrate integrated *J_SC_* values of 22.4, 23.2, and 22.9 mA/cm^2^, respectively. These *J_SC_* values align closely with those obtained using *J–V* measurements, particularly for PSCs incorporating medium and high H_2_O dose‐SnO_x_. The higher *J_SC_* value obtained from EQE measurements compared with that obtained from *J–V* measurements for PSCs incorporating low H_2_O dose‐SnO_x_ further indicates that the PSC performance is limited by the presence of a transport barrier rather than by light absorption or charge‐carrier generation within the perovskite bulk [27]. Since EQE measurements are performed under lower light intensities than the 1‐sun illumination used during *J–V* measurements, the resulting photogenerated charge‐carrier density is significant lower [28]. Under 1‐sun illumination, the higher charge‐carrier density could cause severe charge‐carrier accumulation at the interfacial barrier, which screens the internal electric field and consequently reduces the extracted current, even under short‐circuit conditions.

**FIGURE 5:**
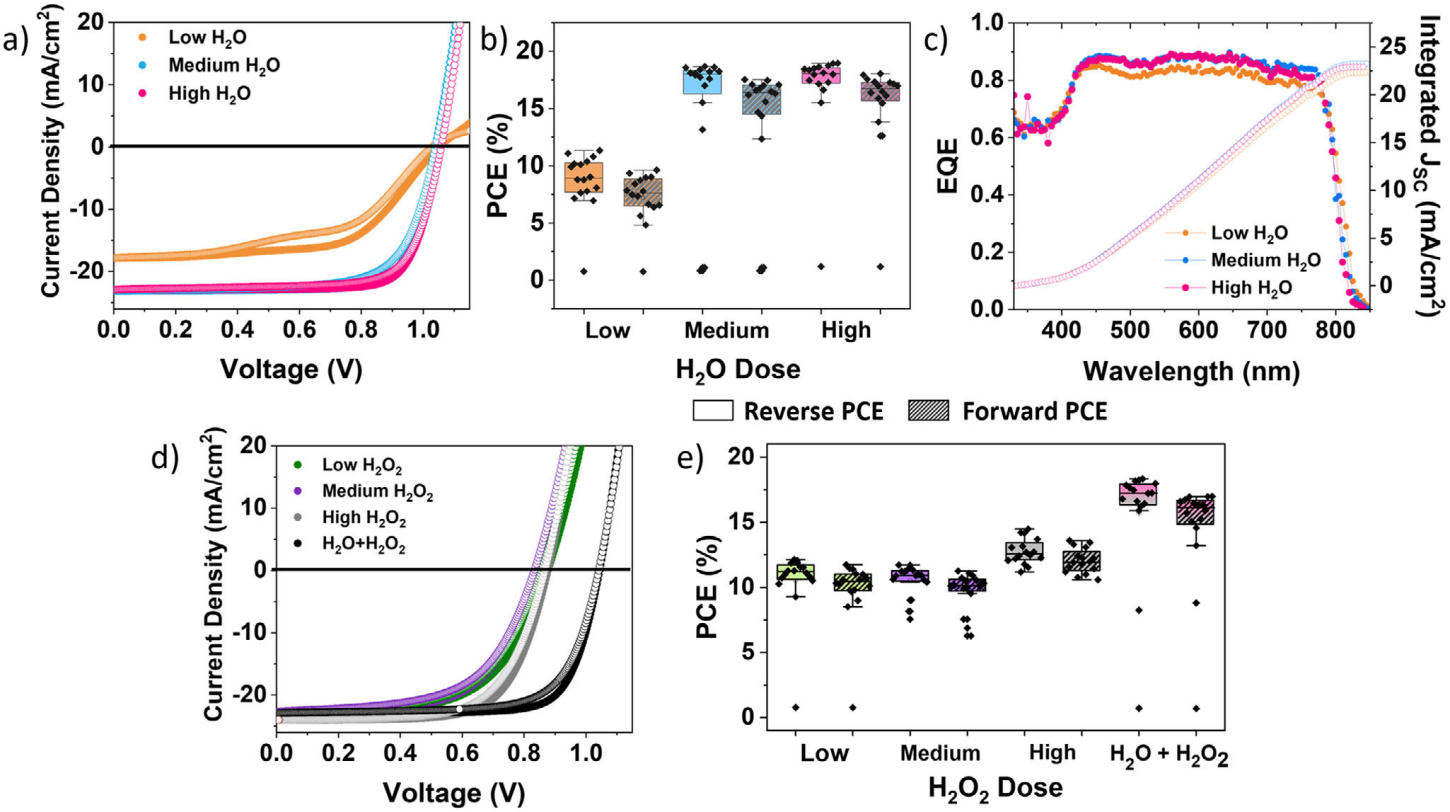
Reverse scan (solid circles) and forward scan (open circles) current‐voltage characteristics of PSCs containing SnO_x_ prepared using different doses of (a) H_2_O and (d) H_2_O_2_ co‐reactants. Box plots represent the effect of (b) H_2_O dose, and (e) H_2_O_2_ dose on the PCE of PSCs. (c) EQE spectra and integrated *J_SC_* values of PSCs presented in (a).

*J–V* characteristics obtained for (Cs, FA)PbI_3_ PSCs comprising SnO_x_ prepared using H_2_O_2_ as co‐reactant are provided in Figure 5d. All PSCs exhibit a considerably lower *V_OC_*, irrespective of the utilized H_2_O_2_ dose, in comparison to PSCs containing H_2_O‐SnO_x_. In addition, the obtained *FF* values are also lower for PSCs containing H_2_O_2_‐SnO_x_ (Figure S13). Nevertheless, the *FF* values of PSCs increase with an increase in the H_2_O_2_ dose utilized during the SnO_x_ process, similar to the case of H_2_O‐SnO_x_. In order to investigate the reason for the lower values of *FF* and *V_OC_* obtained for PSCs comprising H_2_O_2_‐SnO_x_, a combinatory approach was followed. Here, 10 cycles of H_2_O‐SnO_x_ were deposited prior to 170 cycles of H_2_O_2_‐SnO_x_ on top of the C_60_ layer in PSC to minimize the direct contact between H_2_O_2_ and the underlying perovskite/C_60_ structure. As‐fabricated PSCs demonstrate higher PCE, *V_OC_* and *FF* values in comparison to PSC containing H_2_O_2_‐SnO_x_ only. Thereby, the performance of PSCs containing H_2_O_2_‐SnO_x_ is potentially limited by the interface H_2_O_2_‐SnO_x_ forms with the layers underneath rather than its bulk properties.

The effect of co‐reactant utilized during the SnO_x_ ALD was further evaluated on (Cs, FA)Pb(I,Br)_3_ PSCs, with obtained *J–V* characteristics provided in Figure S14. PSCs comprising SnO_x_ prepared using low co‐reactant doses of H_2_O and H_2_O_2_ result in *J–V* characteristics with poor *FF* values. However, the performance of PSCs improves significantly upon utilizing SnO_x_ grown using high doses of both co‐reactants. Thus, the co‐reactant dose utilized during the ALD of SnO_x_ has a similar effect on the performance of both (Cs, FA)PbI_3_ and (Cs, FA)Pb(I,Br)_3_ PSCs. In contrast to (Cs, FA)PbI_3_, (Cs, FA)Pb(I,Br)_3_ PSCs containing H_2_O_2_‐SnO_x_ do not experience a major loss in *V_OC_* in comparison to PSCs employing H_2_O‐SnO_x_. Thereby, it indicates that the interaction between the underlying perovskite composition and H_2_O_2_ preliminary governs the PSC performance, rather than the interaction between C_60_ and H_2_O_2_ only.

Figure S15 presents the photographs of aged (Cs, FA)PbI_3_ PSCs comprising SnO_x_ prepared using different co‐reactants and their respective doses after prolonged storage under low‐humidity conditions. PSC containing SnO_x_ grown using a low dose of H_2_O exhibit rapid degradation of the perovskite phase over time, whereas those containing SnO_x_ grown using a high dose of H_2_O retain the desired black phase. A similar effect is observed for the H_2_O_2_ co‐reactant, as PSCs incorporating SnO_x_ films grown with low co‐reactant doses exhibit faster degradation than those incorporating SnO_x_ films grown with high co‐reactant doses. However, even when a high H_2_O_2_ dose was utilized, degradation was observed to some extent upon prolonged storage. This degradation was fully suppressed when 10 cycles of H_2_O‐SnO_x_ were grown prior to 170 cycles of H_2_O_2_‐SnO_x_. Finally, the PCE values of the non‐encapsulated fresh and aged PSCs incorporating medium and high H_2_O dose‐SnO_x_, as well as a bilayer of H_2_O‐SnO_x_ and H_2_O_2_‐SnO_x_, after prolonged storage under low‐humidity conditions are presented in Figure S15. The samples exhibit only a minor decrease in PCE when evaluated using reverse‐scanned *J–V* characteristics, with the PCE values obtained from forward‐scanned *J–V* measurements remaining essentially unchanged. These results further confirm the long‐term stability and practical relevance of the SnO_x_ ALD process employed in this work.

## 3 Discussion

A clear variation exists in the properties of SnO_x_ as a function of process parameters. The dose of H_2_O co‐reactant employed during the ALD of SnO_x_ impacts the GPC value as well as the concentration of impurities in the film. A gradual increase in the GPC value, during the SnO_x_ ALD as a function of H_2_O dose, is linked to a slow and limited removal of DMA ligands present in the Sn precursor [25, 29]. Higher doses of H_2_O enable better removal of these impurities, as evidenced from RIXS N‐K edge spectra. Simultaneously, the observed increase in the intensity of XPS Sn 3d_5/2_ core‐level peak reveals that the relative concentration of Sn increases with an increase in the H_2_O dose. These observations correlate well with the XRR results that demonstrate an increase in the film density with increasing H_2_O doses. Substitution of more DMA ligands with hydroxy groups, during the H_2_O pulse of the ALD process, provides a larger number of binding sites for TDMASn precursor in the subsequent ALD cycles, and eventually increases the GPC value. Furthermore, the presence of excess ligand impurities and ‐OH groups can be reasoned to create oxide trap charges in low H_2_O dose‐SnO_x_ [30, 31].

In addition to the co‐reactant dose, the employed deposition temperature also affects the GPC value and the film density. The GPC value declines with an increase in the deposition temperature, whereas the film density increases. The O 1s core‐level spectra demonstrate a continuous decrease in ‐OH groups with an increase in the deposition temperature. Previous studies have reported the existence of a similar trend, where the concentration of the reactive hydroxy groups was also found to decrease with an increase in the deposition temperature [14, 25]. Furthermore, as evident from [C]/[Sn] and [N]/[Sn] ratios, the amount of residual DMA ligands in the SnO_x_ films decreases with an increase in the deposition temperature, due to an improved ligand by‐product (H‐DMA) desorption at higher temperatures. Therefore, the combination of higher content of non‐eliminated ligands and presence of more hydroxyl groups at lower temperatures potentially results in higher values of GPC. SnO_x_ films grown at higher deposition temperatures contain a lower amount of bulky organic ligands, thereby allowing the formation of denser and less contaminated SnO_x_ films. Similarly, the higher density values obtained for H_2_O_2_‐SnO_x_ films compared to H_2_O‐SnO_x_ films can be attributed to the lower concentration of hydroxy groups and residual ligand impurities in these films. As previously reported [32], higher GPC values obtained using H_2_O_2_ as the co‐reactant, compared to H_2_O, are associated with the generation of a mixture of reactive hydroxy and oxygen species during the H_2_O_2_ pulse. The greater abundance of these reactive species promotes more efficient ligand removal, thereby creating a large number of surface sites for the adsorption of TDMASn precursor. The difference between the bandgap values of SnO_x_ films prepared using different co‐reactants (H_2_O and H_2_O_2_) is also linked to the variations in the ratio between Sn‐OH and Sn‐O components in the O 1s core‐level spectra. H_2_O_2_‐SnO_x_ exhibits a lower Sn‐OH/Sn‐O ratio compared to H_2_O‐SnO_x_ films at all deposition temperatures. As a consequence, H_2_O_2_‐SnO_x_ films display lower bandgap values in comparison to H_2_O‐SnO_x_ films. In general, films with higher densities and lower Sn‐OH/Sn‐O ratios tend to demonstrate lower bandgap values with higher electron affinities.

(Cs, FA)PbI_3_ and (Cs, FA)Pb(I,Br)_3_ PSCs demonstrate a clear variation in their solar cell performances based on the employed SnO_x_ layer. PSCs comprising high H_2_O dose‐SnO_x_ result in the best PCE values, while the employment of low H_2_O dose‐SnO_x_ leads to s‐shaped *J–V* characteristics. Note that the difference between the thickness values of SnO_x_ layers prepared using different H_2_O doses is too small to affect the solar cell performance. Low H_2_O dose‐SnO_x_ film comprises the highest concentration of DMA ligands among different H_2_O‐SnO_x_ films. The DMA ligands can potentially act insulating and contribute to an increase in the series resistance within the PSC stack. Schottky emission analysis also demonstrates that high H_2_O dose‐SnO_x_ has a slightly higher value of electron affinity (∼3.8 eV) compared to low H_2_O dose‐SnO_x_ (∼3.7 eV). As a consequence, high H_2_O dose‐SnO_x_ might form a slightly better conduction band alignment with the underlying C_60_ layer having an electron affinity of ∼3.98±0.06 eV [33, 34]. Whereas a transport‐barrier might exist at the C_60_/SnO_x_ interface in the case of low H_2_O dose‐SnO_x_. Further, low H_2_O dose‐SnO_x_ contains charge traps, which are absent in high H_2_O dose‐SnO_x_, and high H_2_O_2_ dose‐SnO_x_. These traps are capable of capturing charge carries, and can, therefore, hinder their transport. Increased series resistance, unfavorable conduction band alignment between low H_2_O dose‐SnO_x_ and C_60_, and the existence of traps in low H_2_O dose‐SnO_x_ could potentially introduce a barrier for the extraction of generated charge carriers. Therefore, the employment of low H_2_O dose‐SnO_x_ results in a poor *FF* value and adversely affects the PSC performance. Furthermore, worse stability of PSCs incorporating low H_2_O dose‐SnO_x_ can be explained by an unbalanced charge extraction and existence of excess electrons due to the existence of a transport barrier at the ETL side. Creation of such excess charge carriers have been correlated with a reduced activation energy for ion‐migration, which can promote the perovskite degradation [35, 36].

H_2_O_2_‐SnO_x_ demonstrates higher film density and electron affinity values in comparison to H_2_O‐SnO_x_, which can be expected to result in a better solar cell performance. On the contrary, incorporation of H_2_O_2_‐SnO_x_ layer significantly lowers the *V_OC_* and *FF* values of (Cs, FA)PbI_3_ PSCs. However, (Cs, FA)Pb(I,Br)_3_ PSCs comprising high H_2_O_2_ dose‐SnO_x_ display similar performance as the ones comprising high H_2_O dose‐SnO_x_. Since a C_60_ layer is present in both (Cs, FA)PbI_3_ and (Cs, FA)Pb(I,Br)_3_ PSCs, the possibility of unfavorable interface formation between C_60_ and H_2_O_2_‐SnO_x_ is less likely. Hidalgo et al. studied the impact of combined H_2_O/N_2_ and H_2_O/O_2_ exposures on Cs_0.17_FA_0.83_PbI_3_ using *in situ* grazing‐incidence wide‐angle x‐ray scattering (GIWAXS) and reported that the synergistic effect of H_2_O and O_2_ promotes the transformation of the perovskite phase into undesired 2H and δCs phases [37]. Furthermore, in our previous study, we identified defects at the perovskite/C_60_ interfaces, including openings and local thinning of the C_60_ layer. Since H_2_O_2_ can readily decompose into H_2_O and O_2_, we hypothesize that H_2_O_2_, or its decomposition products, may penetrate through defects in the C_60_ layer or diffuse directly through the bulk of the C_60_ layer and interact with the surface of (Cs, FA)PbI_3_. Such interactions could induce local phase transformations, leading to the formation of surface defects and degradation of perovskite structure. These defects can then act as a potential source of non‐radiative recombination, thus adversely affecting the *V_OC_* and *FF* values of PSCs.

## 4 Conclusion

Process parameters of SnO_x_ ALD play a key role in defining its physical, chemical and electronic properties. In agreement with previous reports, the GPC value and [O]/[Sn] ratio decrease, whereas the film density increases with increasing deposition temperature, irrespective of the employed co‐reactant. Furthermore, H_2_O_2_‐SnO_x_ films exhibit higher density and GPC values in comparison to H_2_O‐SnO_x_ films at all deposition temperatures, which is due to the formation of a mixture of reactive hydroxy and oxygen species during the ALD process employing H_2_O_2_ co‐reactant. Additionally, this work revealed that the dose of the utilized co‐reactant also significantly influences the GPC value and the film density, particularly at 100°C, where both parameters increase with increasing co‐reactant dose. XPS and RIXS clearly demonstrated that higher doses of H_2_O enable better removal of ligand impurities and facilitate a higher Sn concentration within a given volume; thus, ensuring the formation of SnO_x_ films with an improved film density.

The properties of SnO_x_ incorporated in (Cs, FA)PbI_3_ and (Cs, FA)Pb(I,Br)_3_ PSCs substantially influence their performances. PSCs containing high H_2_O dose‐SnO_x_ display the best PCE, i.e., 19%, whereas PSCs comprising low H_2_O dose‐SnO_x_ demonstrate the worst, i.e., 11.3%. High co‐reactant doses are indispensable for achieving efficient solar cell performance as SnO_x_ prepared with low co‐reactant dose results in the formation of a transport barrier between C_60_ and SnO_x_ due its low electron affinity, high series resistance and the presence of charge traps. H_2_O_2_‐SnO_x_ demonstrates worse PCE in comparison to H_2_O‐SnO_x_ when employed in (Cs, FA)PbI_3_ PSC, whereas the PCE values become comparable when incorporated in (Cs, FA)Pb(I,Br)_3_ PSC. Therefore, we hypothesize that H_2_O_2_ might interact differently with (Cs, FA)PbI_3_/C_60_ and (Cs, FA)Pb(I,Br)_3_/C_60_ structures, where its interaction with the former leads to the formation of non‐radiative defect sites.

Our study provides comprehensive insights into the ALD of SnO_x_ for its integration in PSCs, and the role its properties play in influencing the performance of p‐i‐n PSCs. Therefore, this work provides guidance for the optimization of SnO_x_ ALD process with regard to obtaining efficient p‐i‐n PSCs. Further insights on the influence of ALD process parameters on the stability of PSCs and the potential degradation pathways will enable holistic understanding and allow continued advancement of PSCs.

## 5 Experimental Section

Full details of experimental procedures can be found in the Supplemental Information.

## Author Contributions

**Adam Hultqvist**: investigation, writing – review and editing, methodology. **Tobias Törndahl**: investigation, writing – review and editing, funding acquisition, supervision. **Maart Van Druenen**: investigation, methodology, writing – review and editing. **Fredrik Larsson**: investigation, writing – review and editing. **Pontus Törnblom**: investigation, writing – review and editing, data curation, methodology. **Jörgen Olsson**: investigation, writing – review and editing. **Erik M. J. Johansson**: investigation, writing – review and editing. **Erik Wallin**: supervision, resources, investigation. **Carl Hägglund**: investigation, writing – review and editing. **Laurent Duda**: investigation, writing – review and editing. **Evelyn Johannesson**: writing – review and editing, investigation. **Håkan Rensmo**: investigation, writing – review and editing. **Bhavya Rakheja**: conceptualization, investigation, writing – original draft, writing – review and editing, methodology, visualization, formal analysis, data curation.

## Funding

Solar Electricity Research Center, Sweden (SOLVE). Swedish Energy Agency (project number 52693‐1). Myfab Uppsala. Swedish Research Council (2020‐00207),

## Conflicts of Interest

The authors declare no conflicts of interest.

## Supporting information




**Supporting File**: smll75573‐sup‐0001‐SuppMat.docx.

## Data Availability

All supporting results are provided in the Supplemental Information. For further data requests, please contact the corresponding author.
