# Supplementary material for: Investigation of Tin Oxide Atomic Layer Deposition on the Performance of Evaporated Perovskite Solar Cells
Source: Small. 2026 Sep 7;22(62):e75573. doi: 10.1002/smll.75573 (PMC13742896; doi:10.1002/smll.75573)
Supplement: Supplementary file 1 [file SMLL-22-e75573-s001.docx]

**Investigation of Tin Oxide Atomic Layer Deposition on the Performance of Evaporated Perovskite Solar Cells**

Bhavya Rakheja,*^a^ Adam Hultqvist,^a^ Maart Van Druenen,^b^ Pontus Törnblom,^c^ Fredrik Larsson,^b^ Jörgen Olsson,^a^ Carl Hägglund,^a^ Evelyn Johannesson,^c^ Laurent Duda,^c^ Håkan Rensmo,^c^ Erik M. J. Johansson,^d^ Erik Wallin,^b^ and Tobias Törndahl^a^

*^a^Department of Materials Science and Engineering; Solar Cell Technology, Uppsala University, Uppsala, Box 35 SE-751 03, Sweden.*

*^b^First Solar European Technology Center, Vallvägen 5, 756 51 Uppsala, Sweden.*

*^c^Department of Physics and Astronomy; X-ray Photon Science, Uppsala University, Uppsala, Box 516 SE-751 20 UPPSALA.*

*^e^Department of Chemistry - Ångström Laboratory; Physical Chemistry, Uppsala University, Uppsala, Box 523 SE-751 20, Sweden*

Email: bhavya.rakheja@angstrom.uu.se

**EXPERIMENTAL DETAILS:**

***Atomic Layer Deposition (ALD) of SnO_x_***: ALD was performed in Microchemistry F-120 reactor using tetrakis(dimethylamino)tin(IV) (TDMASn) as the tin precursor and deionized water (H_2_O) or hydrogen peroxide (H_2_O_2_), 30% (w/w) in water as co-reactants. The TDMASn bubbler was heated in a water bath at a temperature of 40 ^o^C for at least 1 hour prior to the deposition. Purified N_2_/Ar was used as the carrier gas. An ALD cycle comprised of the sequence TDMASn/N_2_ purge/H_2_O or H_2_O_2_/N_2_ purge, with 0.4 s/0.8 s/X/0.8 s pulse lengths, which was further updated to 0.4 s/1.6 s/X/1.6 s pulse lengths for the SnO_x_ employment in solar cell structures. In combination with pulse length, the co-reactant dose was also varied by changing the number of turns on the needle valves connected to the H_2_O/H_2_O_2_ canisters. In total, three different needle valve and pulse length settings, called low, medium and high, were utilized to vary the co-reactant dose in this study. The table below shows the total dose for the H_2_O source, obtained by measuring the weight of the H_2_O evaporator prior to and after cycling. The amount of evaporated H_2_O_2_ is estimated to be slightly less due to a lower vapor pressure compared to H_2_O.

|  | Total mass change (mg) | Total number of ALD cycles | H_2_O dose (mg/cycle) |
| --- | --- | --- | --- |
| Low | 90 | 15000 | 0.006 |
| Medium | 910 | 15000 | 0.061 |
| High | 1290 | 5000 | 0.258 |

Thin-films were deposited on quartz substrates for spectroscopic ellipsometry (SE), and X-ray reflectivity (XRR) measurements at different deposition temperatures (90 °C, 120 °C, 150 °C, and 180 °C). Simultaneously, SnO_x_ films were also grown on 300 nm thick molybdenum-coated soda lime glass (SLG) substrates for X-ray photoelectron spectroscopy (XPS) and resonant inelastic X-ray scattering (RIXS) measurements. For solar cell structures, 180 ALD cycles of SnO_x_ were grown on top of C_60_ layer at a deposition temperature of 100 °C.

***Solar Cell Fabrication****:*

***Perovskite deposition:*** Perovskite films with composition FA_0.85_Cs_0.15_PbI_2.7_Br_0.3_ and FA_0.93_Cs_0.07_PbI_3_ were deposited from PbI_2_, CsBr, CsI and FAI in a laboratory-scale thermal evaporator on glass substrates coated with fluorine-doped tin oxide (FTO) and a PTAA-based hole-transport layer. After deposition, the films were annealed at 160 °C for 10–20 min on a hot-plate under nitrogen flow.

***C60:*** 12 nm of C60 was thermally evaporated at a deposition rate of 0.3 Å/s. After ALD SnOx, 100 nm indium zinc oxide was deposited by sputtering. Ag metal contacts with a thickness of 135 nm were deposited by evaporation through a shadow mask.

***Metal-Oxide-Semiconductor (MOS) Structures****:* 100-oriented n-type silicon wafers with a resistivity value of 17-25 Ω cm were utilized for the fabrication of MOS structures. Wafers were cleaned using the standard RCA process, and native oxide was removed using a HF dip. As-cleaned wafer was immediately transferred to the ALD reactor and 200/400 cycles of SnO_x_ were grown at a deposition temperature of 100 °C. 500 nm of Al was evaporated at a deposition rate of 5-30 Å/s. The frontside stack was protected by spin coating 1813 resist and baking it at 115 °C for 2 min. The backside oxide was then etched using buffered HF for 8 min. 500 nm of Al was evaporated at a deposition rate of 5-30 Å/s onto the backside of the wafer. Finally, the resist protecting the frontside was removed in a sequence of 60 s, 10 s, and 10 s acetone baths followed by a 1 min 30 s ethanol bath.

***In-situ*** ***Quartz Crystal Microbalance (QCM)***: A 6 MHz gold coated quartz crystal was mounted on the ALD holder to monitor the in-situ growth of SnO_x_. The crystal was connected to an Inficon SQM-160 controller capable of recording the changes in the frequency of the crystal. The change in frequency was then utilized to calculate the change in mass per unit area using the Sauerbrey equation given below:

$$\frac{\Delta m}{A}=-\frac{\sqrt{\rho_{q}\mu_{q}}}{2f_{0}^{2}}\Delta f$$

Here, $\Delta m$ is the change in mass, $A$ is the active area of the crystal, $\Delta f$ is the change in frequency, and $f_{0}$ is the resonant frequency of the crystal. $\rho_{q}$ and $\mu_{q}$ are the density and shear modulus of the quartz crystal, respectively.

***X-ray Diffraction (XRD)***: The measurements were performed in the grazing incidence mode on PANalytical Empyrean setup at an angle of 0.5° between the 2Ѳ range of 5°-70° with a step size of 0.03°.

***X-ray Reflectivity* *(XRR)***: The measurements were conducted on Philips X’pert MRD system. The set-up comprised of an X-ray mirror with a slit size of 1/32° on the incident beam side and a 0.09 parallel plate collimator with a PW3011 (miniprop. small window) detector. All the measurements were performed in the 2Ѳ range of 0.155°-5° with a step size of 0.01°. X’pert Reflectivity software was utilized to simulate the measured curves to obtain film density, thickness and roughness values. Prior to the acquisition of XRR curves, sample height (z), omega (ω) and chi (𝜒) were iteratively aligned to minimize any sample position error. Overall, the error in the density, which is proportional to the square of the critical angle, can be estimated to be less than 5% of the absolute value.

***X-ray Fluorescence* *(XRF)*:** A PANalytical Epsilon5 energy dispersive spectrometer with a Ge detector was used to measure the relative number of tin atoms in the deposited films. For the analysis, the area of the Sn Kα peak was determined from spectra measured with a BaF_2_ secondary target and X-ray tube settings of 85 kV and 6 mA, respectively.

***X-ray photoelectron spectroscopy* (*XPS*)** measurements for studying the effect of H_2_O doses were conducted on a home-built setup comprising a monochromatized Al Kα X-ray source and a hemispherical electron analyzer EW4000 (Scienta Omicron). A base pressure of 1x10^-9^ mbar was maintained in the analysis chamber during the data acquisition. All the core-level spectra were acquired by utilizing a pass energy of 200 eV and an energy step size of 0.1 eV. As-obtained XPS spectra were calibrated by measuring Au foil and fixing the binding energy of Au 4f_7/2_ core-level at 84.0 eV.

*XPS* measurements for comparing the effect of H_2_O and H_2_O_2_ co-reactants were performed on PHI *Quantera* II setup using monochromatized Al Kα X-ray source. The analysis chamber was maintained at an approximate background pressure of 1x10^-9^ mbar. Survey spectra were acquired using a pass energy of 224 eV, dwell time of 50 ms, and step size of 0.8 eV. For the acquisition of detailed core-level spectra, pass energy and step size values were reduced to 55 eV and 0.1 eV, respectively.

In order to fit the core-level spectra, a Shirley background was subtracted and a pseudo-Voigt function was utilized. Elemental concentration ratios of [O 1s]/[Sn 3d_5/2_], [C 1s]/[Sn 3d_5/2_], and [N 1s]/[Sn 3d_5/2_] were calculated from their total integrated peak areas after correcting them with their respective inelastic mean free path (IMFP) and Scofield photoionization cross-section values.^1^ IMFP values were calculated using TPP-2M formula on *‘QUASES-IMFP-TPP2M’* software.^2^

***Resonant inelastic X-ray scattering (RIXS)*** at the N K- and O K- edges was carried out at the iRIXS endstation, beamline 8.0.1, Advanced Light Source (Lawrence Berkeley National Laboratory, Berkeley, CA, USA). All measurements used a slitless variable-line-spacing (VLS) plane-grating spectrograph coupled to a CCD detector, in a vertical scattering geometry with a 160° scattering angle. The beamline provided a resolving power 1228-1938 in the energy range 80-550 eV and an incident photon flux of ~10^12^ photons s^-1^, additional details can be found in Ref.^3^ The incident polarization was horizontal for all experiments. The X-ray spot was ~100 × 100 μm^2^ and was continuously swept across the sample by a small (roughly 1 mm) oscillatory motion of the manipulator during acquisition to average out any inhomogeneities and to mitigate beam damage.

***Current-Voltage (IV) and Capacitance-Voltage (CV) characteristics*** of MOS structures were acquired using HP 41420 A source/monitor unit and HP 4284A precision meter, respectively. Gate bias was applied between the top aluminum contact and the aluminum contact on the back side of Si wafer. *IV* sweeps were performed by changing the voltage from -1 V to 2 V with a step size of 0.075 V. Forward and reverse *CV* curves were acquired from -1 V to 1 V using a step size of 0.1 V.

***Solar cell IV measurements***: J-V curves were measured by a 2450 Keithley source meter. The solar simulator was LED based and from G2V Sunbrick, class AAA. Calibration was carried out to AM1.5G by KG3 filtered silicon cell. Hysteresis scan 1.2 to 0 V and sweep back to 1.2 at a rate of 200 mV/s.

***External quantum efficiency (EQE)*** measurements were performed on a home-built setup comprising xenon lamp coupled to a monochromator. The setup was calibrated using a silicon reference cell prior to the acquisition of data. All the spectra were acquired in the wavelength range of 330 nm-900 nm using a step size of 5 nm. Short-circuit current (*J_SC_*) was calculated by integrating EQE with the standard AM 1.5G spectrum across the measured wavelength range.

Figure S1. QCM curves obtained for 20 ALD cycles of SnO_x_ grown using different H_2_O doses at 100 °C.

Figure S2. Measured and simulated XRR curves of SnO_x_ films deposited on quartz substrates using 1000 ALD cycles and different H_2_O doses at 100 °C.

Table S1. Density and thickness values obtained using XRR, and mass change per unit area values obtained using QCM, for the SnO_x_ ALD process utilizing different H_2_O doses at 100 °C.

| Process | **XRR**  (1000 cycles of SnO_x_ on quartz) | | **QCM** |
| --- | --- | --- | --- |
|  | Density (g/cm^3^) | Thickness (nm) | Mass change, 20 cycles (ng/cm^2^) |
| Low dose | 4.52 | 113 | 1485 |
| Medium dose | 4.86 | 123 | 1628 |
| High dose | 4.99 | 129 | 1667 |

Figure S3. XRF Sn Kα counts measured on SnO_x_ films (1000 ALD cycles) deposited on SLG, and QCM mass change per cycle values obtained for the SnO_x_ ALD process employing different H_2_O doses at 100 °C.

Figure S4. QCM saturation curves obtained for the SnO_x_ process by growing 40 ALD cycles using H_2_O and H_2_O_2_ as co-reactants at deposition temperatures of 90 °C and 150 °C as a function of (a) TDMASn pulse length, (b) co-reactant pulse length and (c) both N_2_ purge lengths.

Figure S5. (a) X-ray diffraction patterns of SnO_x_ films (1000 ALD cycles) deposited on molybdenum-coated SLG substrates, and (b) measured and simulated XRR curves of SnO_x_ films (1000 ALD cycles) deposited on quartz substrates using H_2_O and H_2_O_2_ as co-reactants at different deposition temperatures.

Table S2. Density and thickness values obtained from XRR for SnO_x_ films grown using H_2_O and H_2_O_2_ as co-reactants at different deposition temperatures.

| Process | **XRR**  (1000 cycles of SnO_x_ on quartz) | |
| --- | --- | --- |
|  | Density (g/cm^3^) | Thickness (nm) |
| H_2_O 90 °C | 4.80 | 124 |
| H_2_O_2_ 90 °C | 4.75 | 128 |
| H_2_O 120 °C | 5.23 | 98 |
| H_2_O_2_ 120 °C | 5.40 | 102 |
| H_2_O 150 °C | 5.52 | 76 |
| H_2_O_2_ 150 °C | 5.69 | 77 |
| H_2_O 180 °C | 5.70 | 56 |
| H_2_O_2_ 180 °C | 5.85 | 62 |

**Optical Characterization**

Spectroscopic ellipsometry (SE) was utilized to study how the optical properties vary as a function of the process parameters. Standard ellipsometry measurements were performed in a Woollam RC2 system, for three angles of incidence and 210 to 1690 nm wavelengths. The ellipsometric data was fitted using an optical model comprising a combination of Psemi-M0 and Gaussian oscillator functions, to obtain the SnO_x_ film thickness and dielectric function as a function of wavelength. From this, the refractive index (*n*), extinction coefficient (*k*) and absorption coefficient (*α*), were derived for different SnO_x_ thin-film samples.

Based on the energy dependence of the obtained absorption coefficients, the Tauc method^4^ was adopted to estimate the bandgap value from:

$$\left( \alpha h\upsilon\right)^{\frac{1}{n}} =A(h\upsilon-E_{g})$$

Here, *h* is the Planck’s constant, *υ* is the photon frequency, *A* is a constant and *n* in the exponent depends on the employed material and in particular the type of electronic transition governing the bandgap onset. Considering the amorphous nature of SnO_x_ grown using ALD, *n* = 2 was utilized for the bandgap determination.^4,5^

Figure S6. Bandgap estimation using the Tauc plot for SnO_x_ films (1000 ALD cycles) grown on quartz substrates using (a) different doses of H_2_O at 100 °C, (b) H_2_O and (c) H­_2_O_2_ at different deposition temperatures. UV-Visible transmittance of the same SnO_x_ films grown using (d) H_2_O and (e) H_2_O_2_ at different deposition temperatures. (f) Bandgap values obtained from (b) and (c) as a function of deposition temperature.

Table S3. Optical bandgap values for SnO_x_ films grown using different doses of H_2_O at 100 °C.

| Process | Bandgap (eV) |
| --- | --- |
| Low | 3.22 |
| Medium | 3.26 |
| High | 3.15 |

Table S4. Optical bandgap values for SnO_x_ films grown using H_2_O and H_2_O_2_ at different deposition temperatures.

| Deposition Temperature | Bandgap (eV) | |
| --- | --- | --- |
|  | H_2_O | H_2_O_2_ |
| 90 °C | 3.27 | 3.21 |
| 120 °C | 2.72 | 2.68 |
| 150 °C | 2.63 | 2.59 |
| 180 °C | 2.76 | 2.64 |

**X-ray Photoelectron Spectroscopy**

Figure S7. XPS spectra of O 1s, Sn 3d_5/2_, N 1s and C 1s core-levels of SnO_x_ films (1000 ALD cycles) deposited on molybdenum-coated SLG substrates using different doses of H_2_O at 100 °C.

Figure S8. RIXS N K-edge SnO_x_ films (1000 ALD cycles) deposited on molybdenum-coated SLG substrates using different doses of H_2_O at 100 °C. The energy values indicated in the figure represent the incident excitation photon energy used during the measurements.

Figure S9. (a) RIXS O K-edge spectra and corresponding (b) RIXS maps of SnO_x_ films (1000 ALD cycles) grown on molybdenum-coated SLG substrates using different doses of H_2_O at 100 °C. The energy values indicated in figure (a) represent the incident excitation photon energy used during the measurement.

Figure S10. XPS O 1s, Sn 3d_5/2_, N 1s, and C 1s core-level and valence band (VB) spectra of SnO_x_ films (1000 ALD cycles) grown on molybdenum-coated SLG substrates using H_2_O and H_2_O_2_ as co-reactants at different deposition temperatures.

**Electrical Characterization**

**Schottky Emission Analysis**

Parameters utilized for n-doped silicon are presented below:

| $k$ (eV/K) | 8.62 x 10^-5^ |
| --- | --- |
| $A^{*}$ (A/cm^2^/K^2^) | 1.12 x 10^2^ |
| $T$ (K) | 296 |
| $q$ (C) | 1.60 x 10^-19^ |
| $N_{d}$ (cm^-3^) | 2.50 x 10^14^ |
| $\boldsymbol{\varphi}_{n}$ (eV) | 0.3 |
| $\chi_{Si}$ (eV) | 4.05 |

Here, $k$ corresponds to the Boltzmann constant, $A^{\boldsymbol{*}}$ is the Richardson constant, $T$ represents the temperature, and $q$ is the elementary charge. $N_{d}$, $\varphi_{n}$ and $\chi_{Si}$ represent the doping density, energy difference between the fermi-level and conduction band, and electron affinity of the utilized n-dope silicon.

The barrier height between the fermi-level of n-doped silicon and the conduction band of SnO_x_ can be calculated using equation S1, where b corresponds to the y-intercept of the *ln(J/T^2^)* vs *(E)^1/2^* plot.

$\varphi_{B}\boldsymbol{=}\frac{\boldsymbol{-kT}}{\boldsymbol{q}}\left( b-\ln A^{*} \right)$	(S1)

The obtained barrier height can then be used to calculate the electron affinity of SnO_x_ layer ($\chi_{ox}$) as follows-

$\chi_{ox}=\chi_{Si}\boldsymbol{+}\varphi_{n}- \varphi_{B}$	(S2)

**Capacitance-Voltage Characteristics**

| Parameter | Value |
| --- | --- |
| $N_{d}$ (cm_-3_) | 2.50 x 10^14^ |
| $d$ (nm) | ~20 |
| $\epsilon_{si}$ | 11.7 |
| $\epsilon_{o}$(F/cm) | 8.85 x 10^-14^ |
| $\varphi_{f}$ (eV) | 0.26 |
| $W_{SCR}$ (cm) | 1.65 x 10^-4^ |
| $C_{d}$ (F/cm^2^) | 5.2 x 10^-11^ |

*C-V* characteristics were analyzed using the parameters provided above. $N_{d}$ corresponds to the doping density of silicon, *d* is the thickness of SnO_x_ layer, $\epsilon_{si}$ is the dielectric constant of silicon, $\epsilon_{o}$ is the permittivity of free space, $\varphi_{f}$ is the distance of the fermi level of silicon from its intrinsic level. $W_{SCR}$ is the maximum attainable depletion width in the silicon and $C_{d}$ is the corresponding minimum capacitance, calculated using the following equations.

$W_{SCR}=\sqrt{\frac{4\epsilon_{si}\epsilon_{o}\varphi_{f}}{qN_{d}}}$	(S3)

$C_{d}=\frac{\epsilon_{si}\epsilon_{o}}{W_{SCR}}$ (S4)

The $C_{ox}$ across the SnO_x_ layer was obtained from the accumulation region in the *C-V* characteristics, as indicated in Figure S11b. The obtained values of $C_{ox}$ are 2.1x10^-7^ F/cm^2^ and 1.3 x10^-7^ F/cm^2^ for 20 nm and 40 nm thick SnO_x_ layers, respectively. These capacitance values correspond to a dielectric constant ($\epsilon_{ox}$) value of 5.3 for 20 nm thick SnO_x_ layer and 6.2 for 40 nm thick SnO_x_. Following this, flat-band capacitance ($C_{FB}$) was calculated using the relation (S5).

$\frac{1}{C_{FB}}=\frac{d}{\epsilon_{ox}\epsilon_{o}}+\frac{L_{D}}{\epsilon_{Si}\epsilon_{o}}$ , $L_{D}=\sqrt{\frac{{kT\epsilon}_{Si}\epsilon_{o}}{N_{d}q^{2}}}$ (S5)

Here, $L_{D}$ is the extrinsic Debye length. MOS structure comprising 20 nm and 40 nm thick SnO_x_ layers demonstrate $C_{FB}$ values of 3.46x10^-8^ F/cm^2^ and 3.13 x10^-8^ F/cm^2^, respectively. The corresponding flat-band potential values ($V_{FB}$) for these $C_{FB}$ values are 0.07 V and 0.23 V for MOS structure containing 20 nm and 40 nm of SnO_x_ layer. $V_{FB}$ is determined by the difference between the work-functions of metal and semiconductor ($\varphi_{MS}$) as well as the charges present in the oxide layer as follows:

$V_{FB}= \frac{\varphi_{MS}}{q}-\frac{1}{{qC}_{ox}}(Q_{f}+Q_{OT}+Q_{m})$ (S6)

Here, $Q_{f}$, $Q_{OT}$, and $Q_{m}$ correspond to fixed oxide charge, oxide-trapped charges, and mobile oxide charges, respectively.

**Figure S11.** Measured and simulated *C-V* characteristics of (a) MOS structure containing ~20 nm and ~40 nm thick low H_2_O dose-SnO­_x_ layers. (b) Comparison between *C-V* characteristics of MOS structures containing low H_2_O dose-SnO_x_, high H_2_O dose-SnO_x_ and high H_2_O_2_ dose-SnO_x_.

**Solar Cell Characterization**

**Figure S12.** Current-voltage characteristics of (Cs,FA)Pb(I,Br)_3_ PSCs comprising different number of SnO_x_ ALD cycles. Box plot showing the distribution of PCE values acquired on 16 cells of each variation.

Figure S13. Open-circuit voltage (*V_OC_*), fill factor (*FF*) and short-circuit current (*J_SC_*) values of PSCs comprising SnO_x_ prepared using varying doses of H_2_O and H_2_O_2._

**Figure S14.** Reverse (solid circles) and forward (open circles) *J-V* scans, and box plots showing the distribution of PCE values, for (Cs,FA)Pb(I,Br)_3_ PSCs comprising SnO_x_ prepared using H_2_O and H_2_O_2_ as co-reactants with varying doses.

**Figure S15.** Box plots showing the distribution of PCE values measured for non-encapsulated fresh and aged (Cs,FA)PbI_3_ PSCs incorporating medium and high H_2_O dose-SnO_x_, as well as a bilayer of H_2_O-SnO_x_ and H_2_O_2_-SnO_x_, stored under low-humidity conditions.

**References:**

(1)	Scofield, J. H. *Theoretical Photoionization Cross Sections from 1 to 1500 keV..*; 1973.

(2)	Tanuma, S.; Powell, C. J.; Penn, D. R. Calculations of Electron Inelastic Mean Free Paths. V. Data for 14 Organic Compounds over the 50–2000 eV Range. *Surface and Interface Analysis* **1994**, *21* (3), 165–176. https://doi.org/10.1002/sia.740210302.

(3)	Qiao, R.; Li, Q.; Zhuo, Z.; Sallis, S.; Fuchs, O.; Blum, M.; Weinhardt, L.; Heske, C.; Pepper, J.; Jones, M.; Brown, A.; Spucces, A.; Chow, K.; Smith, B.; Glans, P.-A.; Chen, Y.; Yan, S.; Pan, F.; Piper, L. F. J.; Denlinger, J.; Guo, J.; Hussain, Z.; Chuang, Y.-D.; Yang, W. High-Efficiency in Situ Resonant Inelastic x-Ray Scattering (iRIXS) Endstation at the Advanced Light Source. *Rev. Sci. Instrum.* **2017**, *88* (3), 033106. https://doi.org/10.1063/1.4977592.

(4)	Tauc, J.; Grigorovici, R.; Vancu, A. Optical Properties and Electronic Structure of Amorphous Germanium. *physica status solidi (b)* **1966**, *15* (2), 627–637. https://doi.org/10.1002/pssb.19660150224.

(5)	Klein, J.; Kampermann, L.; Mockenhaupt, B.; Behrens, M.; Strunk, J.; Bacher, G. Limitations of the Tauc Plot Method. *Advanced Functional Materials* **2023**, *33* (47), 2304523. https://doi.org/10.1002/adfm.202304523.
